# The long non-coding RNA LINC01013 enhances invasion of human anaplastic large-cell lymphoma

**DOI:** 10.1038/s41598-017-00382-7

**Published:** 2017-03-22

**Authors:** I-Hsiao Chung, Pei-Hsuan Lu, Yang-Hsiang Lin, Ming-Ming Tsai, Yun-Wen Lin, Chau-Ting Yeh, Kwang-Huei Lin

**Affiliations:** 1 Department of Biochemistry, College of Medicine, Chang Gung University, Taoyuan, Taiwan (R.O.C.); 2 Department of Dermatology, Chang Gung Memorial Hospital, Linkou, Taoyuan, Taiwan (R.O.C.); 3 Department of Nursing, Chang Gung University of Science and Technology, Taoyuan, Taiwan (R.O.C.); 4 Department of General Surgery, Chang Gung Memorial Hospital, Chiayi, Taiwan (R.O.C.); 5 Liver Research Center, Chang Gung Memorial Hospital, Linkou, Taoyuan, Taiwan (R.O.C.); 6 Research Center for Chinese Herbal Medicine, College of Human Ecology, Chang Gung University of Science and Technology, Taoyuan, Taiwan (R.O.C.)

## Abstract

Anaplastic large-cell lymphoma (ALCL) is a rare type of highly malignant, non-Hodgkin lymphoma (NHL). Currently, only studies on the chimeric oncogene NPM-ALK have reported a link to ALCL progression. However, the specific molecular mechanisms underlying the invasion of ALCL are still unclear. Here, we sought to investigate differentially expressed, long non-coding RNAs (lncRNAs) in ALCL and their potential biological function. Our microarray analyses revealed that LINC01013, a novel non-coding RNA gene, was highly expressed in clinical specimens of ALCL and was significantly upregulated in invasive ALCL cell lines. Knockdown of LINC01013 suppressed tumor cell invasion; conversely, its overexpression enhanced tumor cell invasion. LINC01013-induced invasion was mediated by activation of the epithelial-to-mesenchymal transition (EMT)-associated proteins, snail and fibronectin. Specifically, LINC01013 induced snail, resulting in activation of fibronectin and enhanced ALCL cell invasion. Collectively, these findings support a potential role for LINC01013 in cancer cell invasion through the snail-fibronectin activation cascade and suggest that LINC01013 could potentially be utilized as a metastasis marker in ALCL.

## Introduction

ALCL is a distinct subset of non-Hodgkin T-cell lymphoma associated with the presence of large pleomorphic and aberrant cells that express CD30 and T-cell markers^1^. The well known t(2;5)(p23;q35) translocation is the most frequent genetic background in ALCL. This translocation was identified from the resulting fusion of the receptor tyrosine kinase (RTK), anaplastic lymphoma kinase (ALK), with nucleophosmin (NPM)^2^. This NPM-ALK fusion protein, which is highly tyrosine phosphorylated and recruits more than 40 proteins, has been reported to promote ALCL progression by triggering activation of transduction pathways primarily involved in proliferative and antiapoptotic responses^3, 4^. Nevertheless, neither ALK nor other recurrent translocations are expressed in approximately 15–40% of ALCL cases^5^. Although the current World Health Organization classification of lymphomas places ALK(+) and ALK(−) ALCL in the same category, more recent studies suggest that these two types of lymphomas, as well as cutaneous ALCL, might correspond to different entities.

The majority of ALK(+) ALCL cases are diagnosed at advanced stages (III and IV) that display systemic disease with generalized lymphadenopathy and extranodal metastasis, especially in the skin and in soft tissues, such as the liver, lung, and spleen^6^. Similar observations were made in NPM-ALK transgenic mice, confirming that ALK(+) malignancies are very invasive^7^. However, the specific regulatory mechanisms underlying the invasion of ALK(+) ALCL are still unclear.

Recent reports have demonstrated the important role of non-protein–coding RNA (ncRNA) components of the human genome, including microRNAs (miRNAs) and long noncoding RNAs (lncRNAs), in cancer formation or development^8, 9^. Many types of ncRNA play pivotal roles in cancer biology^10, 11^. LncRNAs are aberrantly expressed in various kinds of cancer, and the expression levels of certain lncRNAs are associated with disease progression or diagnosis, or might serve as potential therapeutic targets^12–14^. Further, the clinical significance of the contribution of lncRNAs to ALCL development and the key roles of lncRNA functional networks are currently unknown.

In this study, we investigated the invasion activity involving ALK and the molecular mechanisms underlying the pathogenesis of ALK(+) ALCL. We found that several lncRNA genes were highly expressed in ALCL clinical specimens and hypothesized that their functions were associated with cancer progression. Specifically, our microarray analysis revealed that LINC01013 (long intergenic non-protein–coding RNA 1013), a novel lncRNA that has not been linked to human cancer in the literature, is overexpressed in clinical specimens of ALCL and is significantly up-regulated in invasive ALK(+) ALCL cell lines. We further investigated and characterized this aberrantly expressed lncRNA in ALCL to determine whether it can be used as a novel biomarker and/or therapeutic target for ALCL.

## Results

### Expression of lncRNA in ALCL

**Figure 1:**
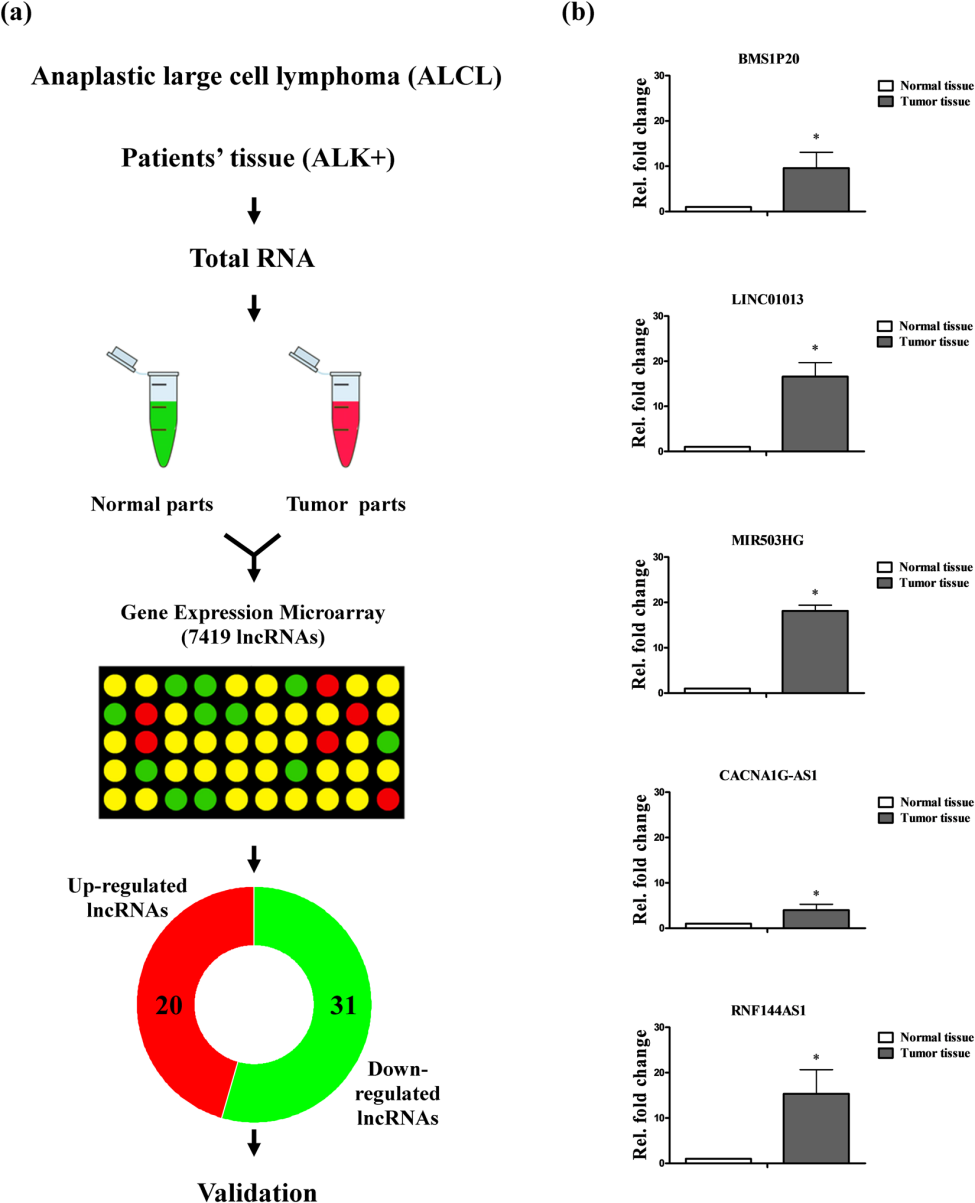
Analysis and validation of lncRNAs in ALCL specimens. (**a**) Schematic diagram showing application of gene expression microarrays (~7419 lncRNAs) to the analysis of lncRNAs. These lncRNAs were selected based on the cut-off value. The 20 up-regulated (fold change > 2.0) and 31 down-regulated (fold change < 0.5) lncRNA were filtered and narrowed down form data base. (**b**) Expression levels of LncRNAs in ALCL specimens (3 paired specimens of patients) were measured by q-RT-PCR. Differences were analyzed using a Kruskal-Wallis test (*P < 0.05).

To identify aberrantly expressed lncRNAs, we characterized lncRNA expression patterns in ALCL specimens using oligonucleotide arrays. Overall, 51 altered lncRNAs were identified in ALCL; 20 were up-regulated and 31 were down-regulated (Fig. 1a). We investigated these overexpressed lncRNAs for their potential function in ALCL progression, focusing on the top five non-coding genes (fold change > 3.5), *BMS1P20, LINC01013, MIR503HG, RNF144A-AS1* and *CACNA1G-AS1* (Table 1). We validated the increased expression of these genes in clinical specimens of ALCL, showing that expression levels were increased ~10-fold for *BMS1P20*, ~17-fold for *LINC01013*, ~19-fold for *MIR503HG*, ~5-fold for *CACNA1G-AS1* and ~15-fold for *RNF144A-AS1* (Fig. 1b). Collectively, these results confirm that these five lncRNAs are highly expressed in ALCL.

**Table 1:** Up-regulated lncRNAs of anaplastic large T cell lymphoma (ALK+).

Gene Symbol	Fold change
BMS1P20	6.707
LINC01013	5.025
MIR503HG	4.419
CACNA1G-AS1	3.712
RNF144A-AS1	3.631
LOC100506027	3.300
MIAT	3.119
FLJ42709	2.767
LOC100506013	2.734
SNORD113-2	2.605
FLJ42709	2.503
MIAT	2.444
LOC339240	2.255
KIAA0125	2.226
MIAT	2.215
MIAT	2.209
MIAT	2.150
SNORD114-11	2.006
SNORD111B	2.004
MIAT	2.001

T/N > 2.0.

### LINC01013 is associated with ALCL cell invasion

**Figure 2:**
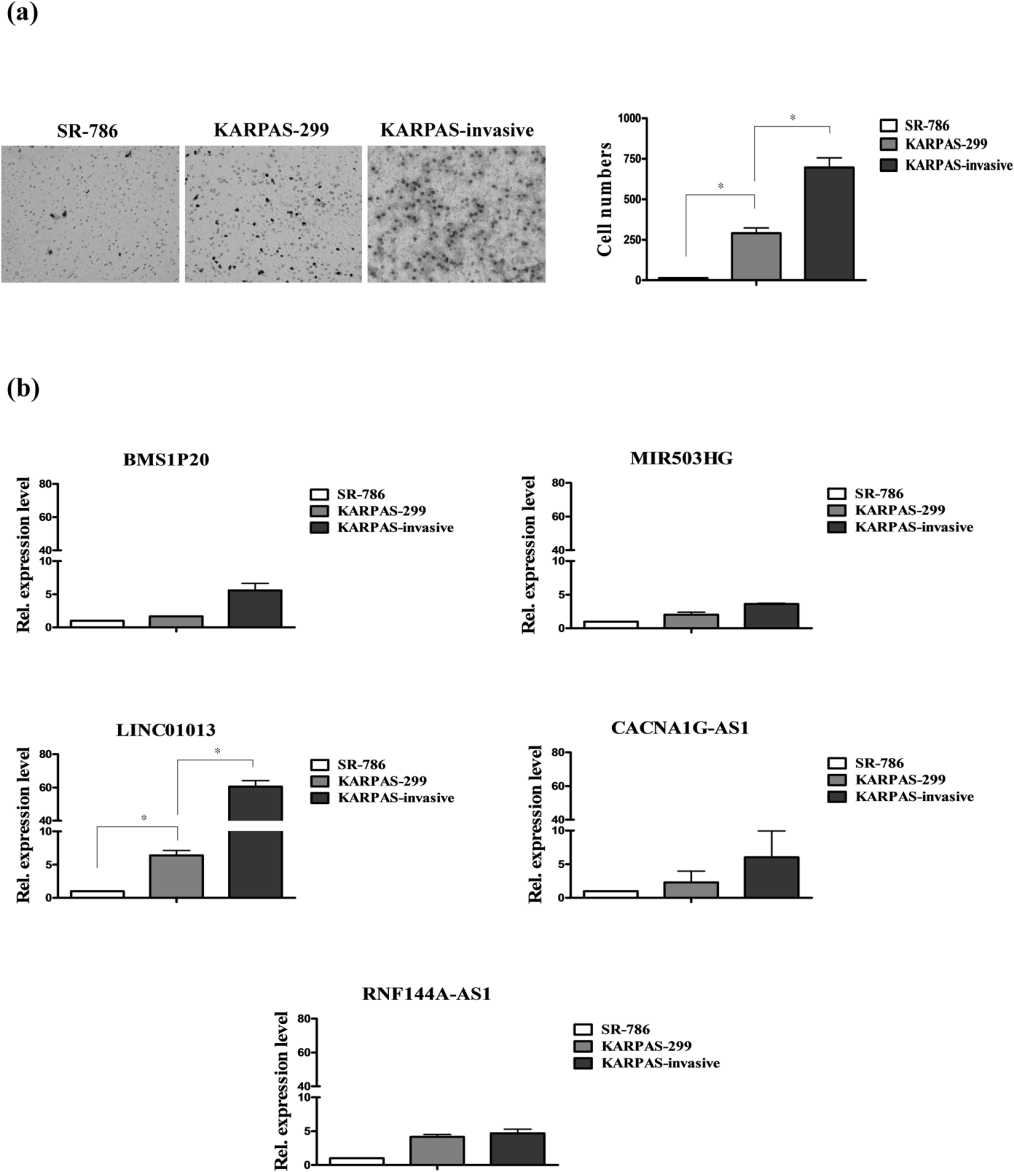
LINC01013 is associated with tumor-invasive functions. (**a**) *Left panel*: Invasion ability was analyzed in SR-786, KARPAS-299, and KARPAS-invasive cell lines using Transwell assays. Invasion activity was determined by counting the number of cells traversing the Matrigel to the lower chamber. Transwell filters were stained with crystal violet. *Right panel*: Quantification of invasion ability. (**b**) BMS1P20, LINC01013, MIR503HG, RNF144A-AS1 and CACNA1G-AS1 expression levels in three cell lines were measured by q-RT-PCR. Differences were analyzed using a Kruskal-Wallis test (*P < 0.05).

To verify the specific functions of lncRNAs in ALCL, we selected three ALK(+) ALCL cell lines: SR-786 (low-invasive), KARPAS-299 (moderately invasive) and Matrigel-selected KARPAS-invasive cells (high-invasive) (Fig. 2a). The expression levels of the lncRNAs, BMS1P20, LINC01013, MIR503HG, CACNA1G-AS1 and RNF144A-AS1 were determined in these cell lines (Fig. 2b). Notably, LINC01013 was highly expressed in the more invasive KARPAS-invasive cell line (Fig. 2b, left panel), suggesting that LINC01013 may be played a pivotal role in ALCL cell invasion.

### LINC01013 depletion suppresses ALCL cell invasion

**Figure 3:**
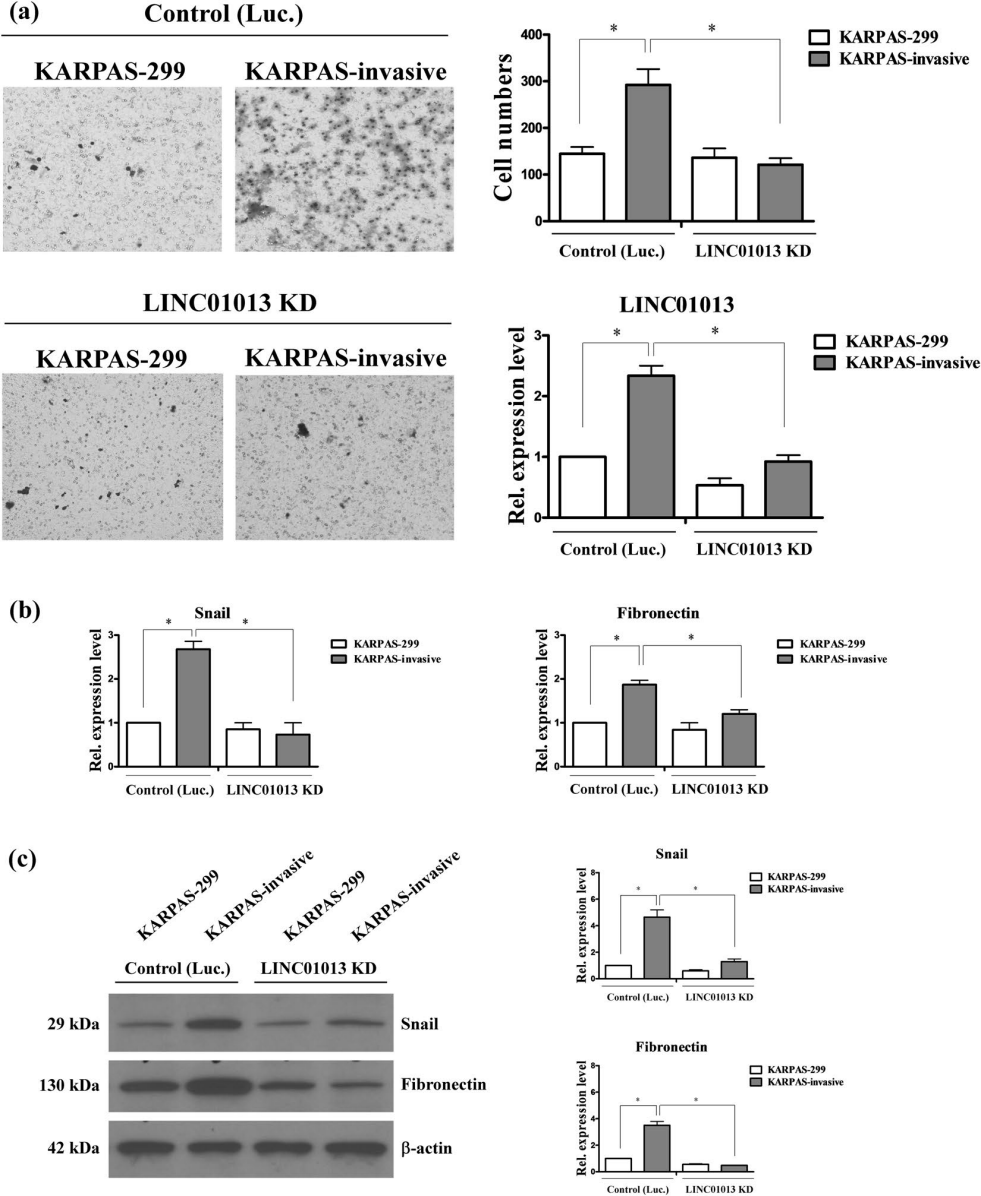
LINC01013 depletion suppresses ALCL cell invasion. (**a**) *Left panel*: The invasion ability of KARPAS-299 and KARPAS-invasive cell lines was analyzed using Transwell assays under LINC01013-depleted (LINC01013 KD) and control (Luc.) conditions. *Right panel*: Quantification of invasive ability and LINC01013 expression levels. (**b**) Snail and fibronectin expression levels in these cell lines were determined by q-RT-PCR (**c**) and Western blot analysis. Differences were analyzed using a Kruskal-Wallis test (*P < 0.05).

**Figure 4:**
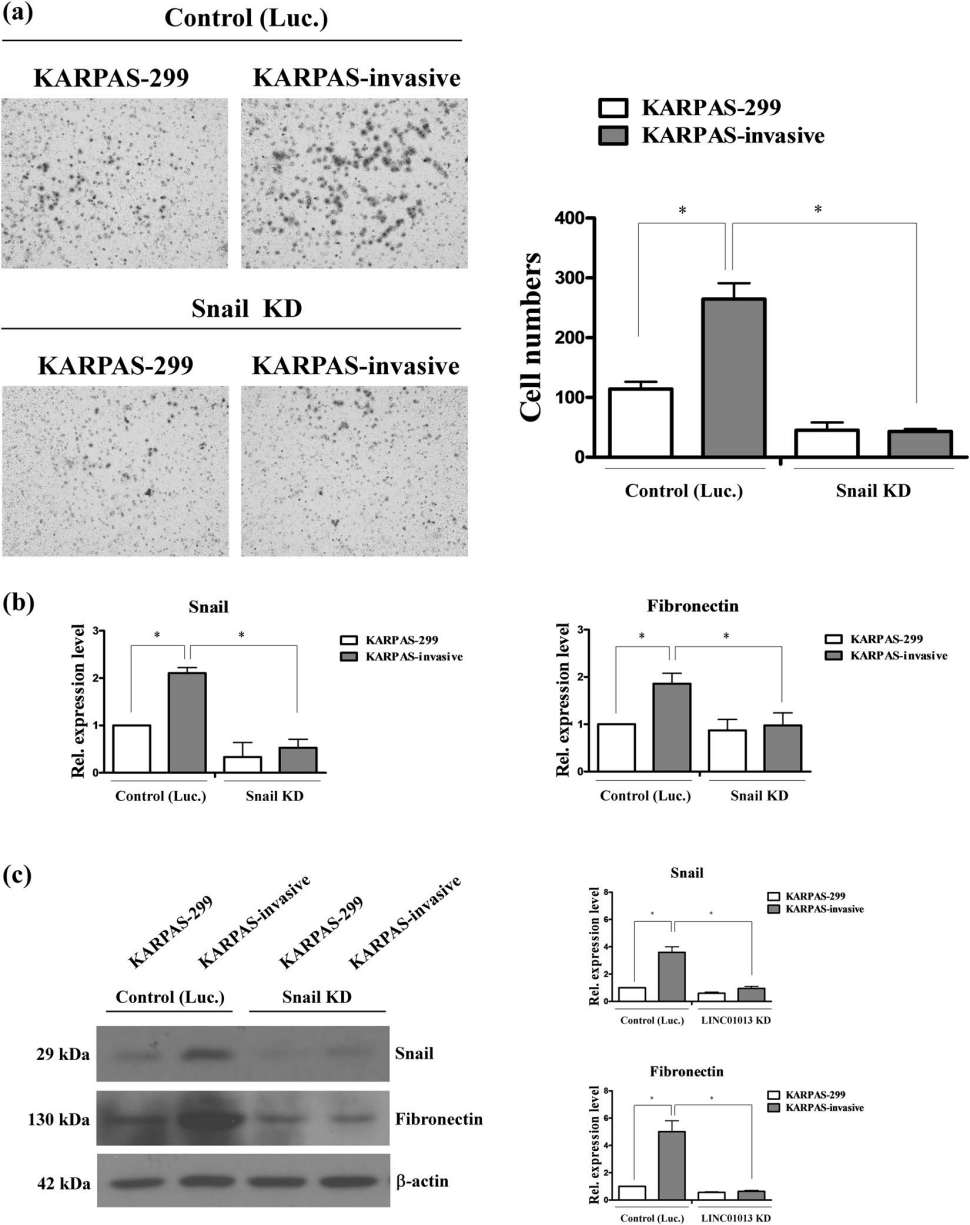
Snail depletion suppresses ALCL cell invasion. (**a**) *Left panel*: Invasion ability of KARPAS-299 and KARPAS-invasive cell lines was analyzed using Transwell assays under snail-depleted (Snail KD) and control (Luc.) conditions. *Right panel*: Quantification of invasion assay results. (**b**) Snail and fibronectin expression levels in these cell lines were determined by q-RT-PC (**c**) and Western blot analysis. Differences were analyzed using a Kruskal-Wallis test (*P < 0.05).

To determine the effects of LINC01013 on cell invasion, we established LINC01013-knockdown KASPAS-299 and KASPAS-invasive cell lines. Notably, KASPAS-299 and KASPAS-invasive cell lines depleted of LINC01013 displayed significantly decreased invasion (~2-fold) compared with control cells (Fig. 3a). The epithelial-to-mesenchymal transition (EMT)-associated markers, snail and fibronectin, were also significantly decreased in LINC01013-depleted cells compared with control cells (Fig. 3b,c). Snail, a transcription factor associated with cancer metastasis, regulates fibronectin to promote lymphoma cell invasion. Similarly, depletion of snail in KASPAS-299 and KASPAS-invasive cells decreased invasion ability compared with control cells (Fig. 4a), and markedly down-regulated fibronectin (Fig. 4b,c). The expression levels of other Snail targets or EMT regulators have no effect between control and snail-depleted cells (Fig. S1) or control and LINC01013-depleted cells (Fig. S2). Taken together, these data confirm the requirement of LINC01013 to accelerate tumor cell invasion and implicate the snail-fibronectin cascade in this process.

### LINC01013 promotes ALCL cell invasion by activating the snail pathway

**Figure 5:**
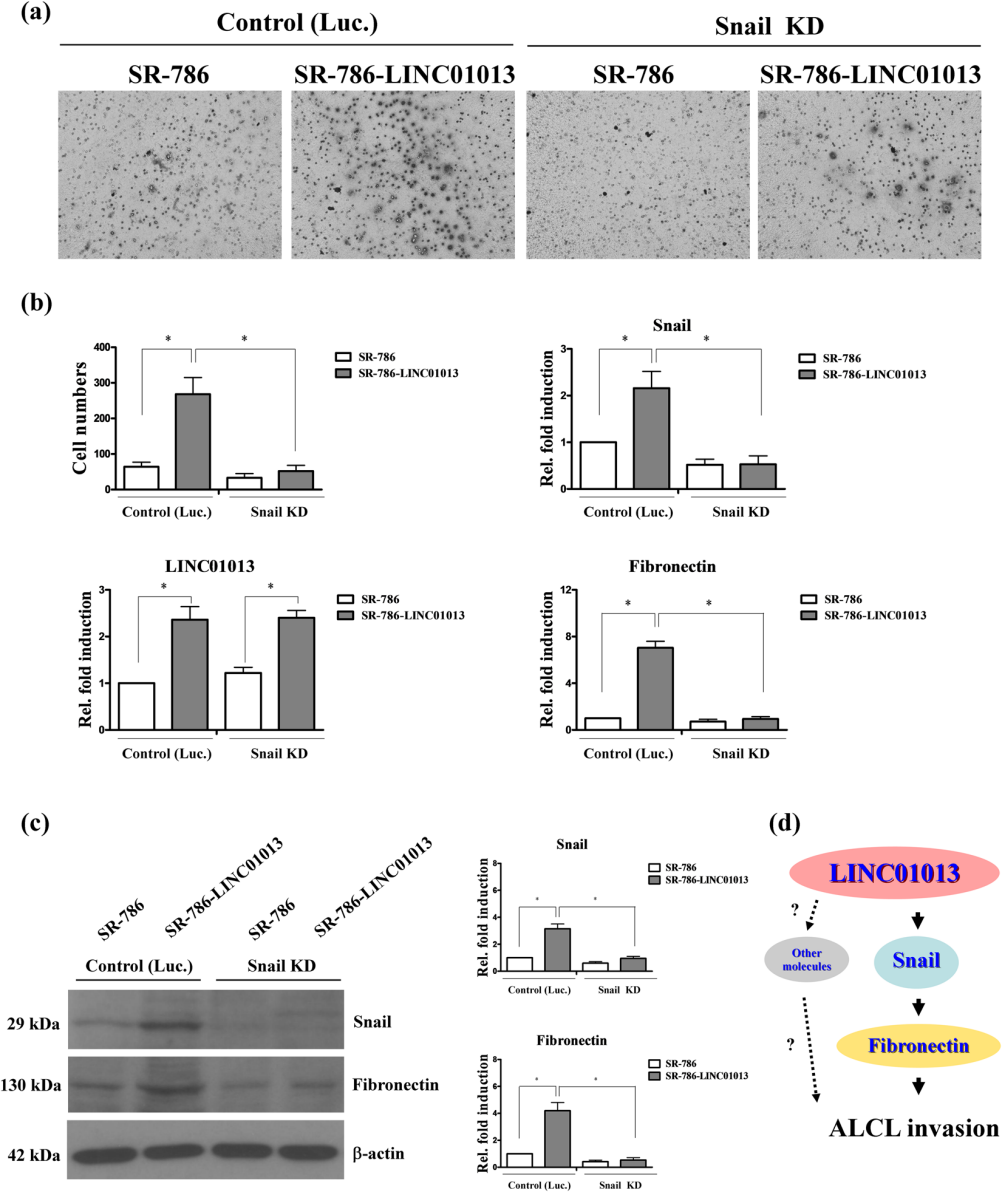
LINC01013 acts through the snail-fibronectin cascade to enhance cell invasion. (**a**) The invasion ability of SR-786 and SR-786-LINC01013 cells was analyzed using Transwell assays under snail-depleted (Snail KD) and control (Luc.) conditions. (**b**) *Left panel*: Quantification of invasive ability and LINC01013 expression levels. *Right panel*: Snail and fibronectin expression levels in these cell lines were determined by q-RT-PCR (**c**) and Western blot analysis. (**d**) Schematic depiction of LINC01013 promotion of ALCL invasion through activation of the snail-fibronectin cascade. Differences were analyzed using a Kruskal-Wallis test (*P < 0.05).

To further confirm the involvement of snail activation in LINC01013-induced phenotypic changes, we performed invasion assays using LINC01013-overexpressing cells. Notably, LINC01013-overexpressing SR-786 cells (SR-786-LINC01013) displayed significantly increased invasion (~2–3-fold) compared with parental SR-786 cell controls (Fig. 5a,b, left panel), and this phenotype was attenuated by snail depletion (Fig. 5a, right panel). SR-786-LINC01013 cells also exhibited marked upregulation of snail and fibronectin, an increase that was specifically blocked by depletion of snail (Fig. 5b, right panel, and c). Collectively, these results support a potential role of LINC01013 in promoting cancer cell invasion through activation of the snail-fibronectin cascade in ALCL (Fig. 5d).

## Discussion

No recent studies have reported an association between lncRNAs and ALCL progression or, by extension, the involvement of an lncRNA-mediated mechanism in the invasion of ALCL. To identify specific pathways underlying ALCL carcinogenesis, we used an oligonucleotide microarray analysis of ncRNA genes to identify lncRNAs that were differentially expressed in ALCL. We further evaluated clinical specimens for aberrantly expressed lncRNAs to identify potential biomarkers of ALCL invasion. Notably, we found that the novel lncRNA, LINC01013, is highly expressed in ALCL specimens and showed that its expression is positively correlated with the invasivity of ALK(+) cells. Gain- and loss-of-function experimental strategies clearly demonstrated that LINC01013 enhanced ALK(+) ALCL cell invasion. Thus, LINC01013 may be a novel oncogene that could serve as an invasion marker for ALK(+) ALCL metastases.

LncRNAs are important regulators of gene expression and are thought to have a wide range of functions in cellular and developmental processes^16–18^. The functions of a few lncRNAs have been experimentally defined^19, 20^. These studies have demonstrated the involvement of lncRNAs in fundamental processes of gene regulation, including chromatin modification, direct transcriptional regulation, RNA processing, post-translational regulation of protein activity or localization, and miRNA modulation. In the current study, we demonstrated that LINC01013 promotes an increase in mRNA and protein expression levels of snail and fibronectin, indicating the involvement of lncRNA in the transcriptional regulation of downstream genes.

The mesenchymal marker snail is a core transcription factor in the EMT process that stimulates expression of the downstream target gene encoding fibronectin to enhance cancer invasion^21^. To date, few studies have linked the regulation of lncRNA and snail/fibronectin in lymphoma. LINC01013 enhances invasion abilities in ALK(+) ALCL cell lines and may stimulate metastasis through activation of snail-fibronectin components. Our findings are consistent with the conclusion that activation of these mesenchymal markers leads to ALCL cell metastasis.

Previous studies of ALCL progression have focused on NPM1-ALK translocation and its relative contribution to pathogenesis^22^. Fusions of ALK have oncogenic potential because their aberrant tyrosine kinase activity enhances cell proliferation and survival and promotes cytoskeletal earrangement^23^. NPM1-ALK–interacting molecules ultimately lead to the activation of key pathways, including RAS/ERK (extracellular signal-regulated kinase), phospholipase C (PLC-γ), phosphoinositide 3-kinase (PI3K), and JAK/STAT (signal transducer and activator of transcription) pathways^24, 25^. Activation of STAT3 is associated with a specific signature that includes several transcription factors (e.g., CEBP/β), cell cycle (e.g., cyclin D, c-myc) and survival/apoptosis molecules (e.g., Bcl-A2, Bcl-XL, survivin, MCL-1), and cell-adhesion proteins^26^. These studies have demonstrated the regulation of coding genes and their cross-talk in ALCL tumor formation. Several reports have shown that miRNAs, a type of short non-coding RNA, regulate ALCL malignancies. For example, miR-29a expression was found to modulate apoptosis through inhibition of MCL-1 expression in ALCL^27^; ectopic expression of miR-150 inhibits proliferation and blocks S-phase entry of ALK(+) cells^28^; and downregulation of miR-16 induces vascular endothelial growth factor (VEGF) expression in ALK(+) ALCL^29^. However, the detailed role of long non-coding genes in the pathogenesis of ALCL is still unclear and may represent an important avenue for future research.

In conclusion, this study is the first to demonstrate the involvement of an lncRNA in ALCL tumor progression. The results provide new insights into the mechanism by which the lncRNA, LINC01013, contributes to the promotion of ALCL cell invasion, showing that it acts through activation of the snail-fibronectin cascade. Our findings collectively support a potential role of LINC01013 in ALCL progression and suggest that LINC01013 expression could be effectively utilized as a metastatic marker in ALCL.

## Materials and Methods

### Ethics statement

All the experiments were performed in accordance with the approved guidelines of the Chang Gung Memorial Hospital Institutions Review Board (IRB: 103-3205B). Informed consent was obtained from all patients involved in this study.

### Tissue specimens

A total of ALCL tumor tissue and adjacent noncancerous mucosa were obtained from Department of Dermatology, Chang Gung Memorial Hospital.

### RNA extraction and gene expression microarrays

Total RNA from paired ALCL tumor tissue and adjacent noncancerous mucosa samples (N = 3) were extracted using the TRIzol reagent (Life Technologies, Rockville, MD, USA), as described previously^15^. Total RNA (20 μg) was used for labeling and hybridization with the SurePrint G3 Human Gene Expression array (Agilent, Welgene Biotech, Taiwan) containing 7419 lncRNAs and 27958 human genes. Slides were scanned and intensities were acquired using GenePix Pro 4.1 software (Axon Instruments Inc. Foster City, CA, USA).

### Cell culture

SR-786, KARPAS-299, and Matrigel-selected KARPAS-invasive human ALK(+) ALCL cell lines were routinely cultured at 37 °C in a humidified atmosphere of 95% air and 5% CO_2_ in Dulbecco’s modified Eagle’s medium (DMEM) supplemented with 10% or 15% fetal bovine serum (FBS). The references for SR-786 and KARPAS-299 cell lines are *Human Cell Culture Vol. III*, *355–370* (*J.R.W. Masters and B.O. Palsson*). These are human ALK(+) large T cell lymphoma cell lines. KASPAS-invasive cells were harvested form three rounds of Matrigel-selected (invasion assay) KASPAS-299.

### Cloning of *LINC01013*

cDNA was synthesized from total RNA (1 μg) using Superscript II reverse transcriptase (Invitrogen, Carlsbad, CA, USA) and random hexamer primers (Invitrogen). *LINC01013* was amplified by polymerase chain reaction (PCR) using the primer pair 5′-CAG GAA GCC AGC ATT TTT AAT-3′ (forward) and 5′-CAA ATA ATA TCT TGC TTT TAT-3′ (reverse), and the following thermocycling conditions: 30 cycles at 95 °C for 1 min, 58 °C for 1 min, and 72 °C for 2 min. The *LINC01013* open reading frame was ligated into the pcDNA 3.0 expression vector, and the resulting construct was sequenced to confirm the presence of the gene.

### Immunoblot analysis

Total cell lysates and conditioned media were prepared, and protein concentrations were determined using a Bradford assay kit (Pierce Biotechnology, Rockford, IL, USA). Equivalent amounts of protein were fractionated by sodium dodecyl sulfate-polyacrylamide gel electrophoresis (SDS-PAGE) on a 10% gel. Separated proteins were transferred to a nitrocellulose membrane (pH 7.9; Amersham Biosciences Inc., Piscataway, NJ, USA), blocked with 5% non-fat powdered milk, and incubated with specific anti-snail (Cell Signaling Technology, Cell Signaling, Danvers, USA; #6615) and anti-fibronectin (Santa Cruz Biotechnology, Santa Cruz, CA, USA; sc-9068) primary antibodies at 4 °C overnight. After washing, membranes were incubated with horseradish peroxidase (HRP)-conjugated anti-mouse, anti-rabbit or anti-goat IgG secondary antibody, as appropriate, for 1 h at room temperature. Immune complexes were visualized using an enhanced chemiluminescence (ECL) detection kit (Amersham) and Fuji X-ray film.

### Establishing SR-786 cell lines stably overexpressing LINC01013

The SR-786 cell line, grown in 10-cm cell culture dishes, was transfected with the LINC01013 expression plasmid using the Lipofectamine reagent (Invitrogen). After 24 h, transformants were selected from transfected cells by growing in medium containing the antibiotic neomycin G418 (400–800 μg/ml) for 2–3 weeks. Survival clones were pooled and used to western blot or functional assay. Expression levels of LINC01013 RNA in the selected clones were determined using quantitative reverse transcription-PCR (q-RT-PCR).

### shRNA-mediated LINC01013 knockdown

Short hairpin RNA (shRNA) sequences targeting LINC01013 were purchased from the National RNAi Core Facility (Institute of Molecular Biology, Academia Sinica, Taiwan). The KARPAS-299 and KARPAS-invasive cell lines were transiently transfected with shRNA targeting the endogenous *LINC01013* gene using the TurboFect reagent (Invitrogen). LINC01013 repression was confirmed by q-RT-PCR.

### shRNA-mediated snail knockdown

Short hairpin RNA (shRNA) sequences targeting snail were purchased from the National RNAi Core Facility (Institute of Molecular Biology, Academia Sinica, Taiwan). The SR-786, SR-786-LINC01013, KARPAS-299 and KARPAS-invasive cell lines were transiently transfected with shRNA targeting endogenous snail mRNA using the TurboFect reagent (Invitrogen). Snail repression was confirmed by Western blot analysis.

### Invasion assays

The influence of LINC01013 on invasion ability *in vitro* was determined by Transwell assay (Falcon BD, Franklin Lakes, NJ) using LINC01013-depleted KARPAS-299 or LINC01013-overexpressed SR-786 cells, as described previously^15^. Briefly, cell density was adjusted to 10^5^ cells/ml, and 100 μl of the suspension was seeded into upper chambers of the Transwell plate, either coated (invasion) or not coated (migration) with Matrigel (Becton-Dickinson). For both assays, the pore size of the upper chamber was 8 mm. The medium in the upper chamber was serum-free DMEM, and the lower chamber contained DMEM supplemented with 20% FBS, included as a chemoattractant. After incubation for 24 h at 37 °C, cells traversing the filter from the upper to lower chamber were stained with crystal violet and counted. Experiments were repeated at least three times.

### Statistical analysis

Data are expressed as mean values ± SEM of at least three experiments. Statistical analyses were performed using Student’s *t* test and one-way analysis of variance (ANOVA). Where appropriate, the Mann–Whitney *U* test or Fisher’s exact test was used to compare two groups; a Kruskal–Wallis test or Pearson’s χ^2^ test was used if more than two groups were compared. The relationship between the results of two different examinations was analyzed with Spearman’s correlation test. *P-*values < 0.05 were considered statistically significant.

## Electronic supplementary material

Supplementary figure legends
