# Supplementary material for: The long non-coding RNA LINC01013 enhances invasion of human anaplastic large-cell lymphoma
Source: Sci Rep. 2017 Mar 22;7:295. doi: 10.1038/s41598-017-00382-7 (PMC5428265; doi:10.1038/s41598-017-00382-7)
Supplement: Supplementary file 1 — Supplementary figure legends [file 41598_2017_382_MOESM1_ESM.pdf]

The long non-coding RNA LINC01013 enhances invasion of human anaplastic large-cell lymphoma

I-Hsiao Chung[1*], Pei-Hsuan Lu[1, 2*], Yang-Hsiang Lin[1], Ming-Ming Tsai[3, 4], Yun-Wen Lin[1], Chau-Ting Yeh[5] and Kwang-Huei Lin[1, 5]

[1]Department of Biochemistry, College of Medicine, Chang Gung University, Taoyuan, Taiwan, R.O.C.

[2]Department of Dermatology, Chang Gung Memorial Hospital, Linkou, Taoyuan, Taiwan, R.O.C.

[3]Department of Nursing, Chang Gung University of Science and Technology, Taoyuan, Taiwan, R.O.C.

[4]Department of General Surgery, Chang Gung Memorial Hospital, Chiayi, Taiwan, R.O.C.

[5]Liver Research Center, Chang Gung Memorial Hospital, Linkou, Taoyuan, Taiwan, R.O.C.

*These authors contributed equally to this work

To whom correspondence and reprint requests should be addressed: Dr. Kwang-Huei Lin, Department of Biochemistry, Chang-Gung University, 259 Wen-hwa 1 Road, Taoyuan, Taiwan, Republic of China (R.O.C). Tel./Fax: +886-3-2118263. E-mail: khlin@mail.cgu.edu.tw

The downstream genes of snail

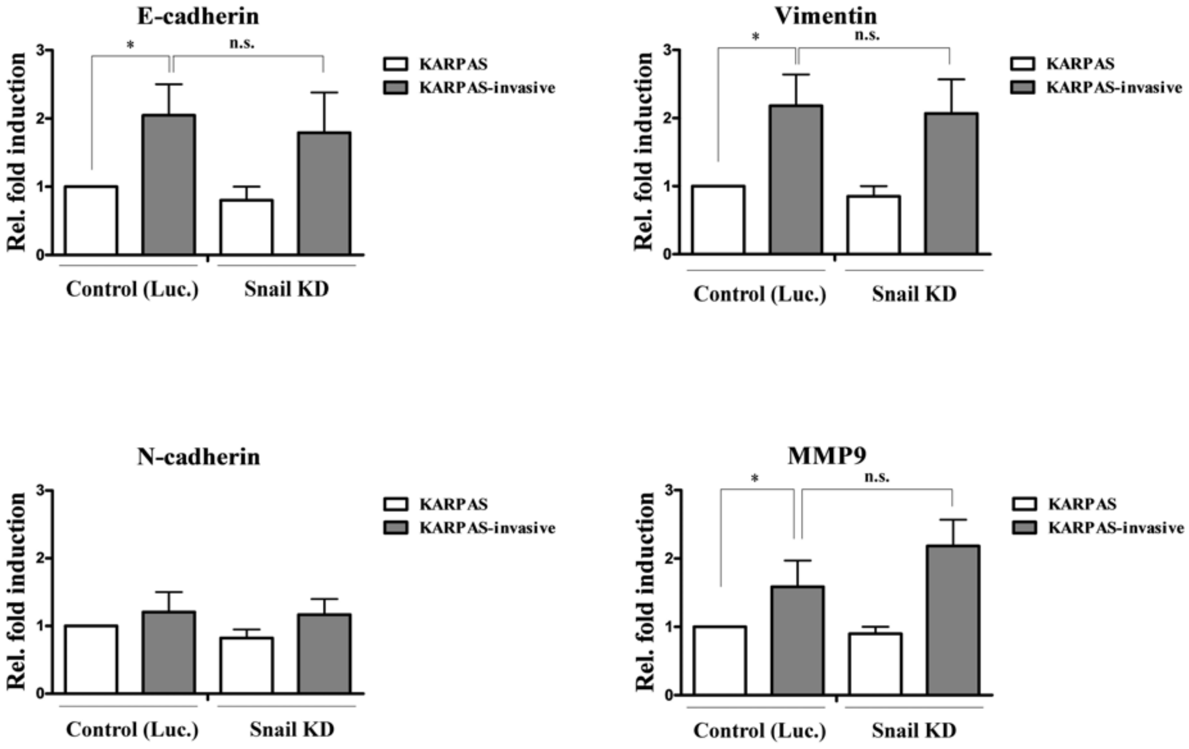

Fig. S1 The downstream genes of snail

E-cadherin, Vimentin, N-cadherin and MMP-9 expression levels in KARPAS-299 and KARPAS-invasive cell lines under snail-depleted (Snail KD) and control (Luc.) conditions were measured by q-RT-PCR. Differences were analyzed using a Kruskal-Wallis test (*$P < 0.05$).

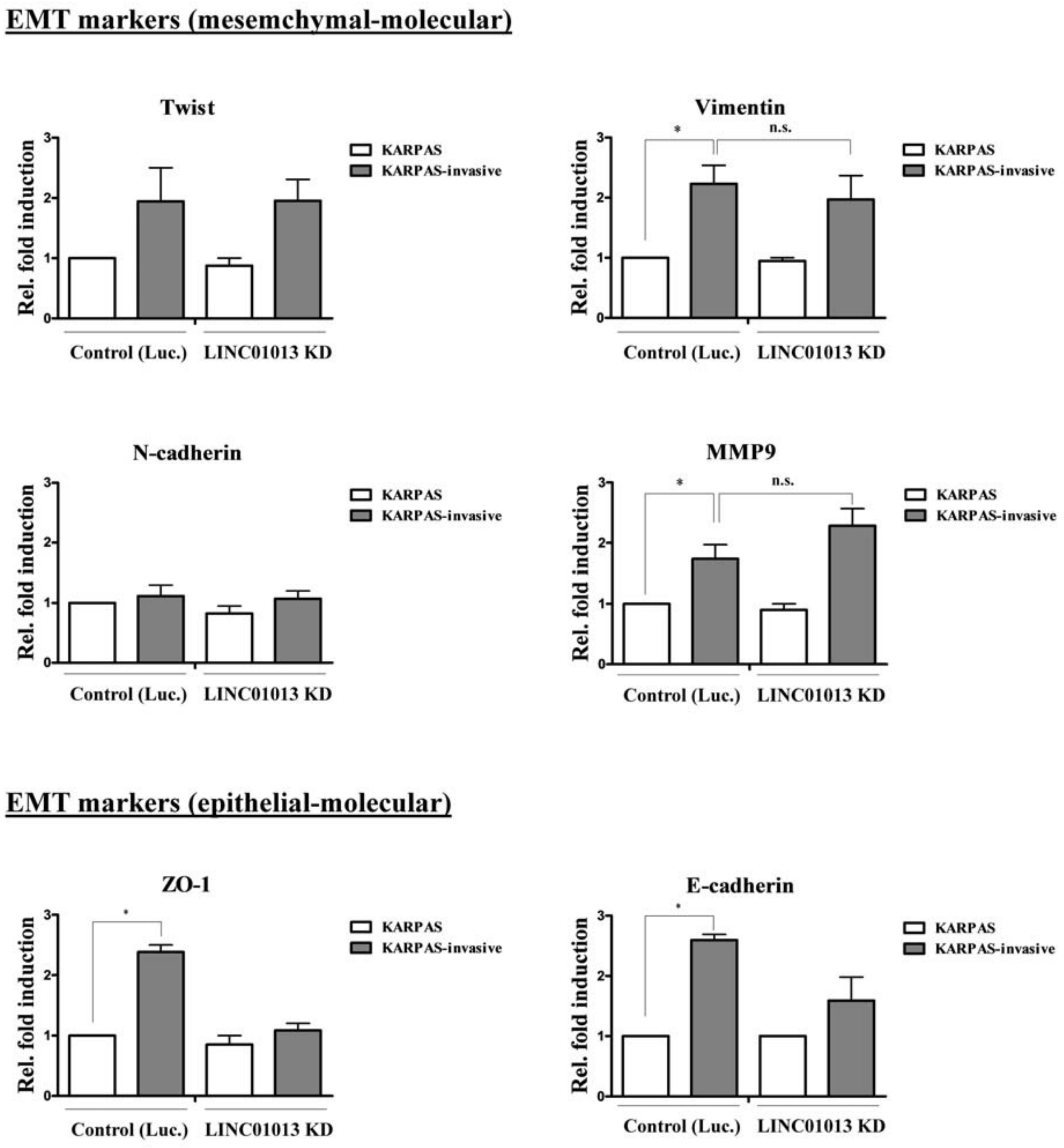

Fig. S2 The expression of other EMT regulators

Twist, Vimentin, N-cadherin, MMP-9, ZO-1 and E-cadherin expression levels in KARPAS-299 and KARPAS-invasive cell lines under LINC01013-depleted (LINC01013 KD) and control (Luc.) conditions were measured by q-RT-PCR. Differences were analyzed using a Kruskal-Wallis test (*$P < 0.05$).

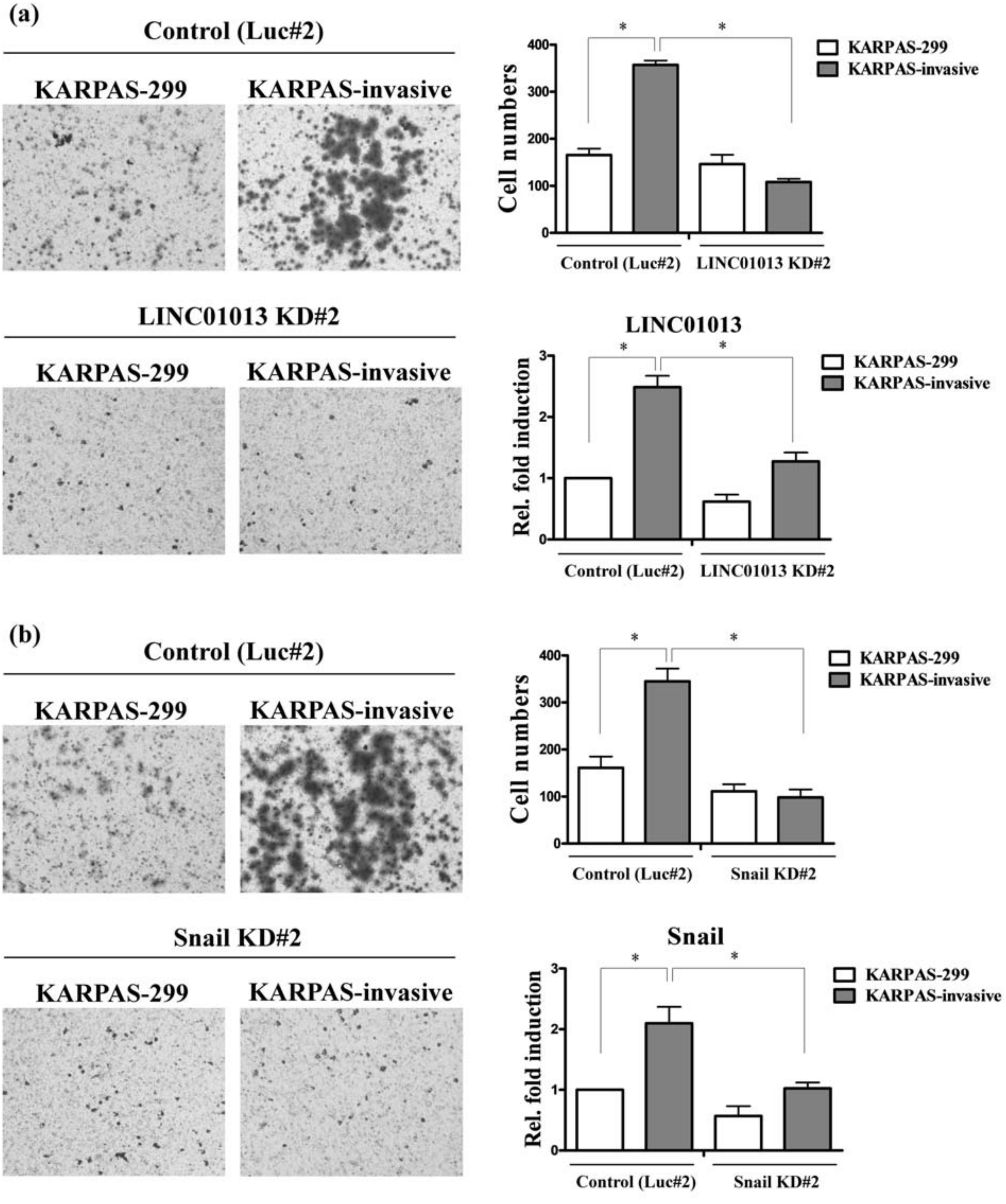

Fig. S3 LINC01013 and Snail depletion suppress ALCL cell invasion

(a) *Left panel:* The invasion ability of KARPAS-299 and KARPAS-invasive cell lines

was analyzed using Transwell assays under LINC01013-depleted (LINC01013 KD#2) and control (Luc#2) conditions. *Right panel:* Quantification of invasive ability and LINC01013 expression levels. (b) *Left panel:* Invasion ability of KARPAS-299 and KARPAS-invasive cell lines was analyzed using Transwell assays under snail-depleted (Snail KD#2) and control (Luc#2) conditions. *Right panel:* Quantification of invasion assay results and snail expression levels. Differences were analyzed using a Kruskal-Wallis test (*$P < 0.05$).

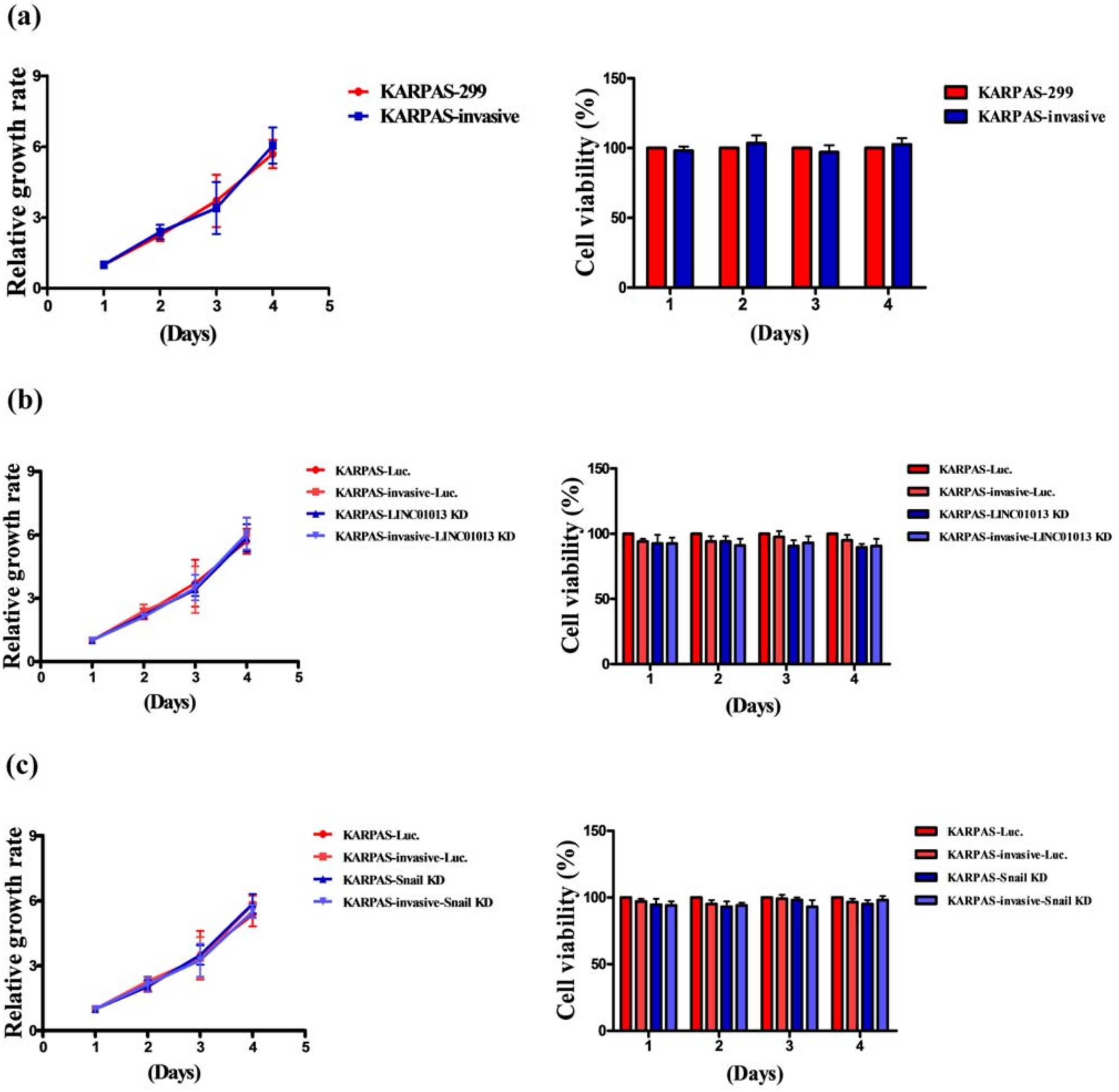

Fig. S4 Cell proliferation and viability of ALCL cell lines

The cell growth ability and viability of KARPAS-299 and KARPAS-invasive cell lines (a); under LINC01013-depleted (LINC01013 KD) (b) and snail-depleted (Snail KD) (c) and control (Luc.) conditions were analyzed using MTT assays.

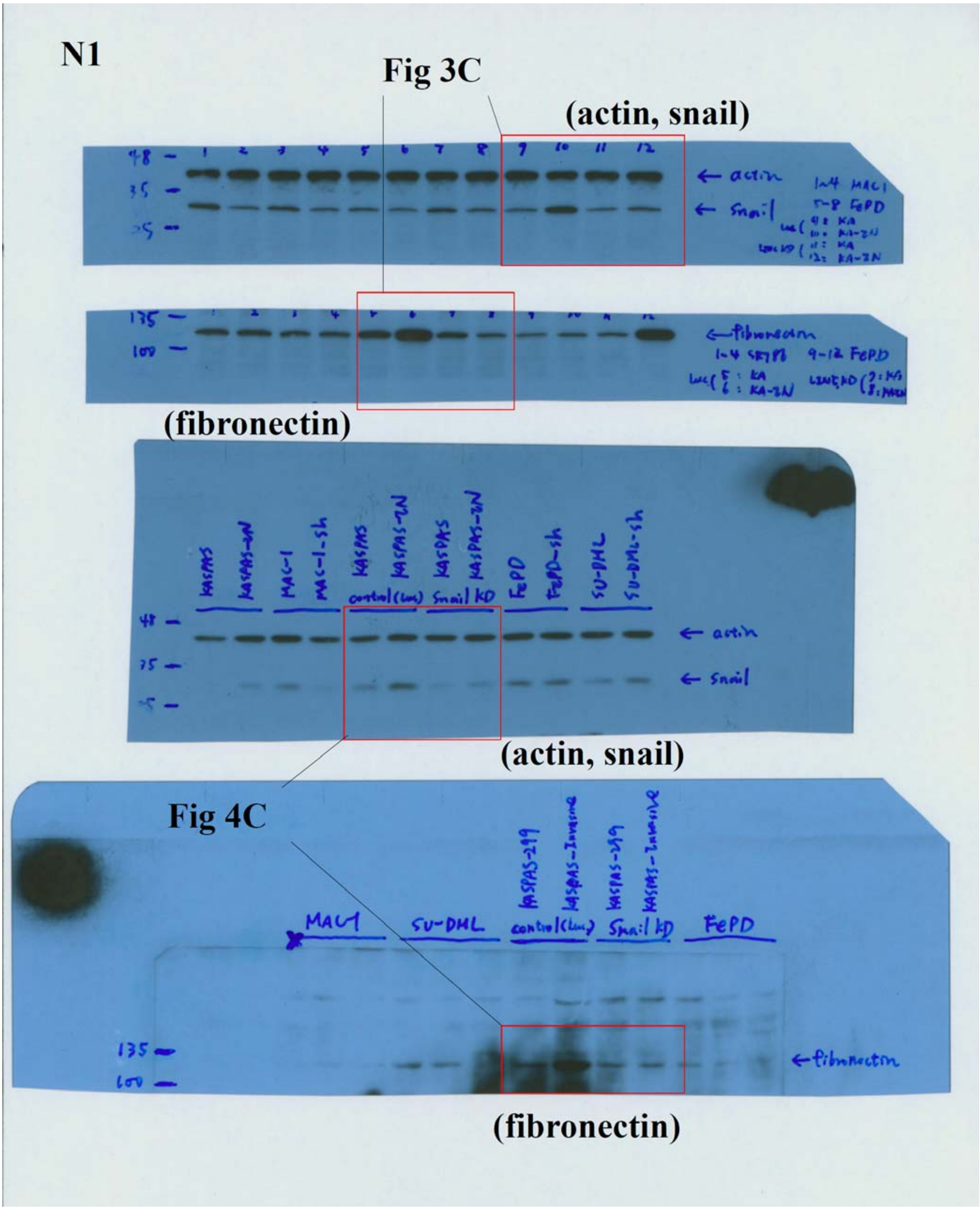
N1
Fig 3C
(actin, snail)
(fibronectin)
(actin, snail)
Fig 4C
(fibronectin)

N1

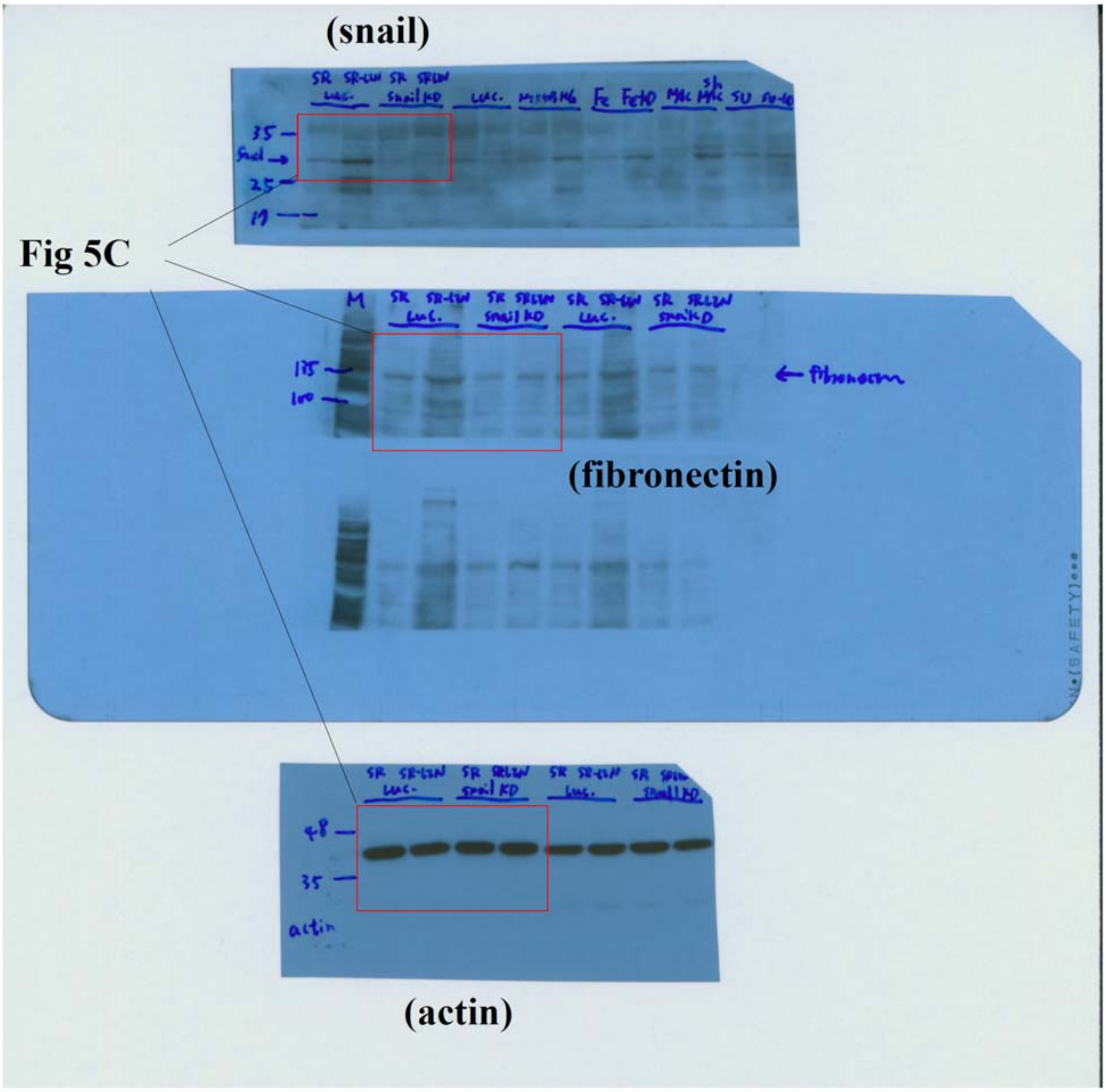

Fig 3C

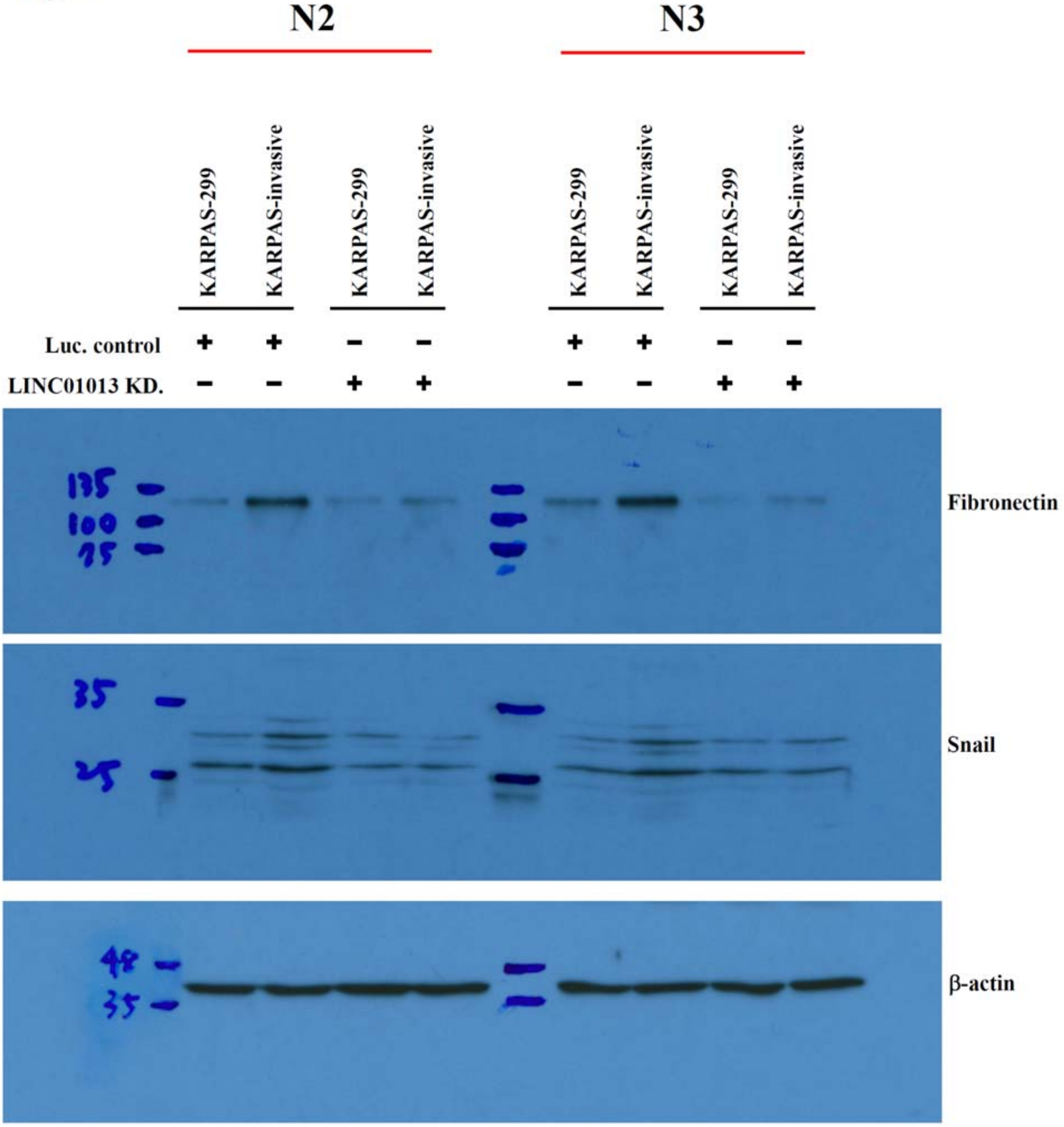

N2
N3
KARPAS-299
KARPAS-invasive
KARPAS-299
KARPAS-invasive
KARPAS-299
KARPAS-invasive
KARPAS-299
KARPAS-invasive
Luc. control + + − − + + − −
LINC01013 KD. − − + + − − + +
135
100
75
Fibronectin
35
25
Snail
48
35
β-actin

Fig 4C

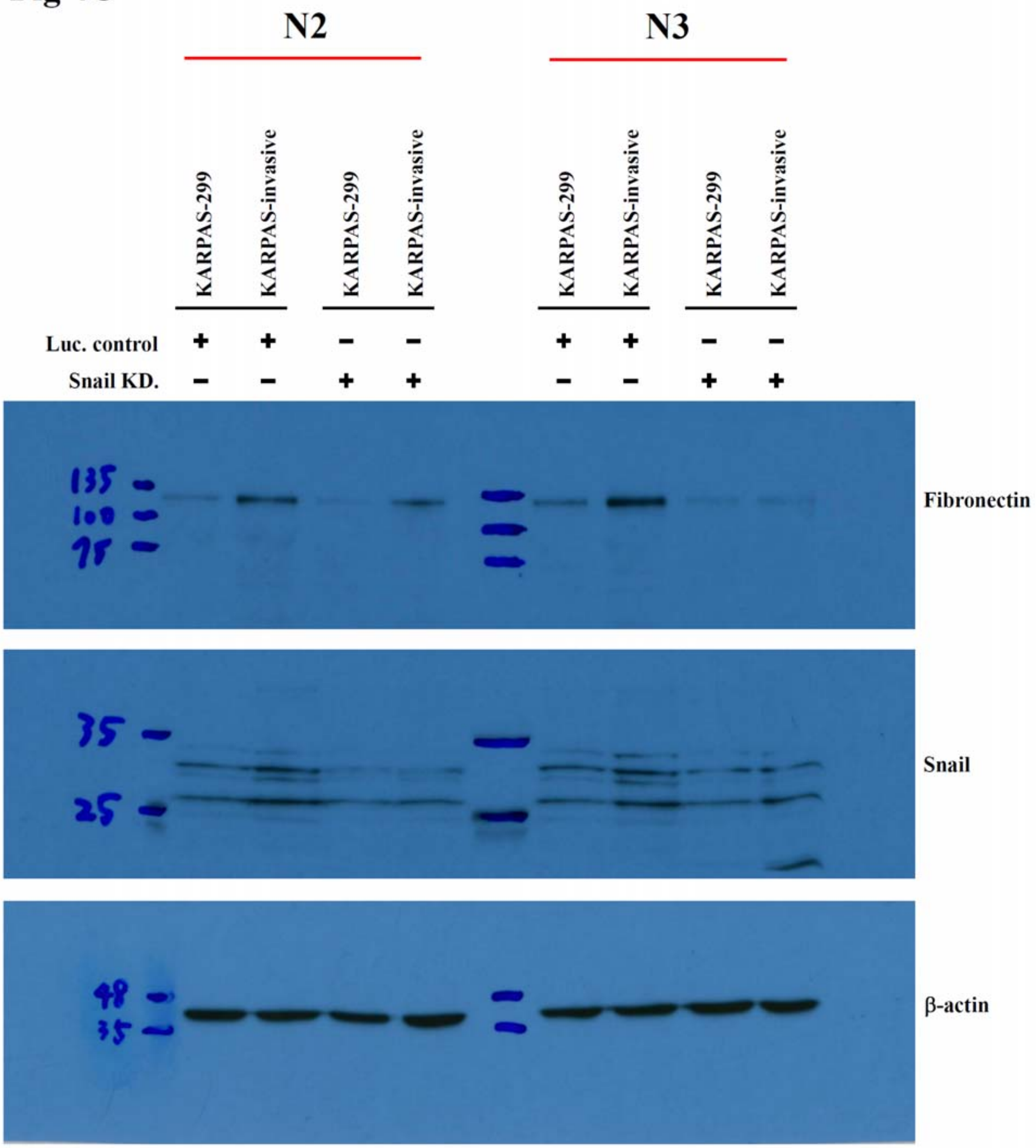
N2
N3
KARPAS-299
KARPAS-invasive
KARPAS-299
KARPAS-invasive
KARPAS-299
KARPAS-invasive
KARPAS-299
KARPAS-invasive
Luc. control + + − − + + − −
Snail KD. − − + + − − + +
135
100
75
Fibronectin
35
25
Snail
48
35
β-actin

Fig 5C

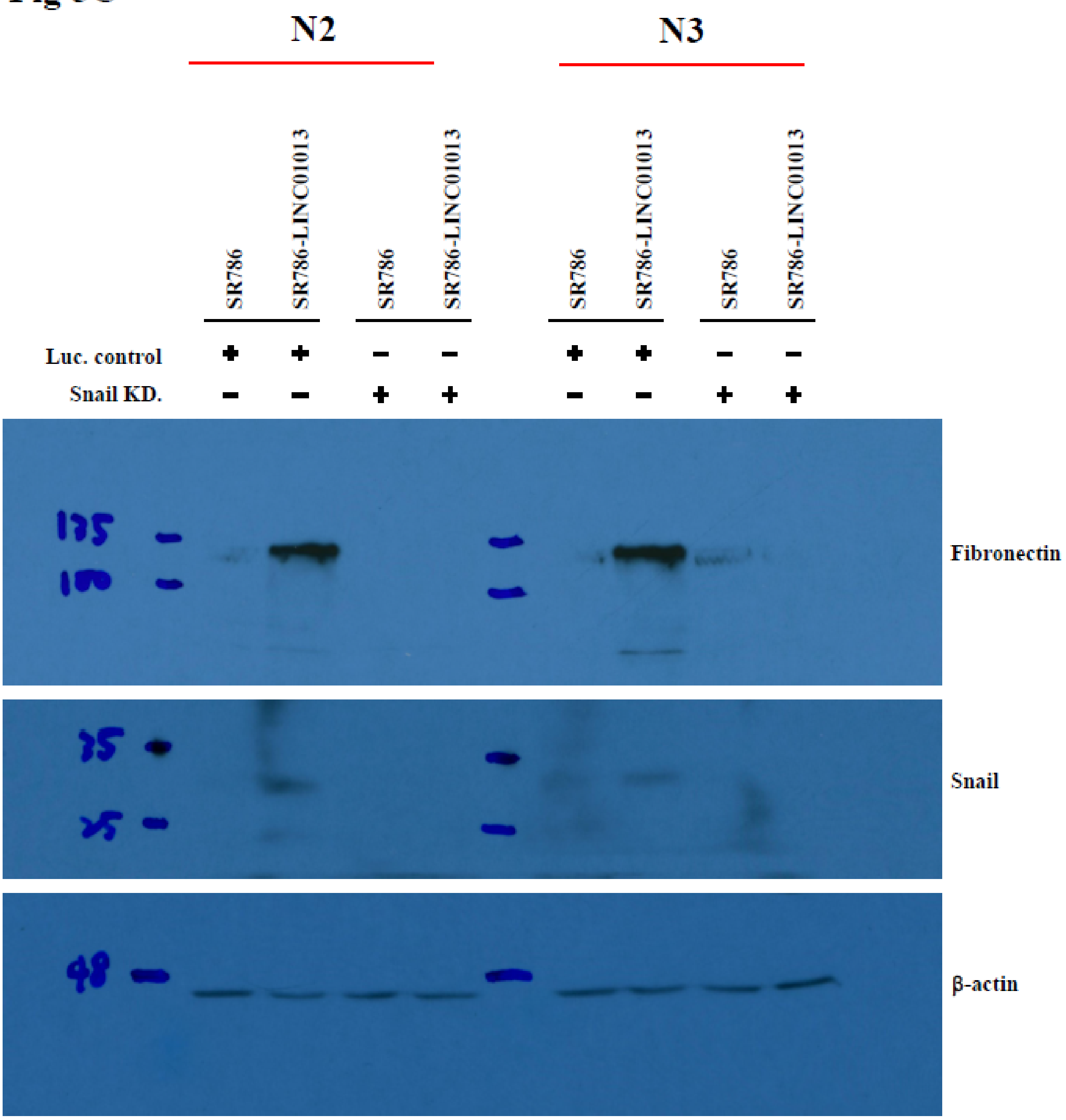
N2
N3
SR786
SR786-LINC01013
SR786
SR786-LINC01013
SR786
SR786-LINC01013
SR786
SR786-LINC01013
Luc. control
Snail KD.
Fibronectin
Snail
β-actin
135
100
35
25
48
